# Extended Intrapleural Tissue Plasminogen Activator (tPA)-Dornase Alfa (DNase) Therapy Beyond the Standard Multicenter Intrapleural Sepsis Trial 2 (MIST-2) Regimen in a High-Risk Patient With Complex Empyema: A Case Report

**DOI:** 10.7759/cureus.107447

**Published:** 2026-04-21

**Authors:** Rodrigo Furlan Silva Fabri, Rodolfo Myronn De Melo Rodrigues, Dan L Zhou, Eshani Kishore, Poornachandran Mohankumar, Maria Opina, Dan Schuller, Patrick Kicker

**Affiliations:** 1 Internal Medicine, Texas Tech University Health Sciences Center El Paso, Paul L. Foster School of Medicine, El Paso, USA; 2 Pulmonary and Critical Care Medicine, Texas Tech University Health Sciences Center El Paso, Paul L. Foster School of Medicine, El Paso, USA; 3 Internal Medicine/Pulmonary and Critical Care Medicine, Texas Tech University Health Sciences Center El Paso, Paul L. Foster School of Medicine, El Paso, USA

**Keywords:** case report, dornase alfa, empyema, intrapleural fibrinolytic therapy, mist-2, tpa

## Abstract

Pleural infection, including complicated parapneumonic effusion and empyema, remains a major management challenge, especially in patients who are poor surgical candidates. The Multicenter Intrapleural Sepsis Trial 2 (MIST-2) supports a six-dose regimen of intrapleural tissue plasminogen activator (tPA) plus dornase alfa (DNase), but the optimal duration of therapy in patients with persistent loculations remains uncertain. We describe a 62-year-old man with morbid obesity, New York Heart Association class III diastolic heart failure, and poorly controlled type 2 diabetes mellitus who presented with a complex, loculated left-sided empyema caused by *Gemella sanguinis* and *Escherichia coli*. Because of prohibitive operative risk, he was not considered a candidate for video-assisted thoracoscopic surgery. After incomplete improvement following the standard six-dose regimen, characterized by persistent fever, persistently elevated inflammatory markers, and residual loculations on repeat imaging, intrapleural therapy was extended for an additional six doses, for a total of 12 doses over six days. The patient had marked clinical and radiographic improvement, complete lung re-expansion, and no bleeding complications. This case suggests that, in carefully selected high-risk patients with persistent loculated empyema and no surgical option, extended intrapleural tPA-DNase therapy may be a feasible individualized strategy beyond the fixed MIST-2 protocol.

## Introduction

Pleural infection encompasses complicated parapneumonic effusion and empyema and is associated with substantial morbidity, prolonged hospitalization, and frequent need for invasive intervention [1-3]. Complicated parapneumonic effusion refers to an infected pleural effusion in which bacterial invasion and pleural inflammation promote fibrin deposition, septation, and loculation, often impairing drainage; empyema represents frank purulence or established pleural space infection [1-3]. Progressive pleural inflammation can therefore limit drainage and complicate source control [1,2]. Current management centers on antibiotics, pleural drainage, and escalation to intrapleural fibrinolytic therapy or surgery when needed [2,3].

The Multicenter Intrapleural Sepsis Trial 2 (MIST-2) established combined intrapleural tissue plasminogen activator (tPA) and dornase alfa (DNase) as the standard non-surgical regimen, using twice-daily administration for three days for a total of six doses [4]. In that trial, combination therapy improved radiographic clearance and pleural drainage compared with placebo or either agent alone [4]. Consensus guidance and subsequent reviews support this approach; however, they do not provide clear guidance on extension beyond the standard six-dose course in patients with persistent loculations or prohibitive surgical risk [1,2,5,6]. We report a high-risk patient with complex empyema who underwent a full second monitored course of intrapleural tPA-DNase, for a total of 12 doses, after an incomplete response to the standard regimen.

This case was presented as a poster presentation at the American Thoracic Society (ATS) International Conference in San Francisco, CA, in May 2025. This article was previously posted to the Research Square preprint server on January 16, 2026.

## Case presentation

A 62-year-old man with morbid obesity (body mass index > 40 kg/m²), New York Heart Association class III diastolic heart failure, and poorly controlled type 2 diabetes mellitus was transferred from an outside hospital after unsuccessful initial management of a progressive left pleural effusion. Given his severe cardiometabolic comorbidity burden and limited physiologic reserve, he was considered a high-risk candidate for general anesthesia and operative intervention. Baseline serum and pleural fluid laboratory results are summarized in Table 1. On arrival, he had severe dyspnea, cough, and hypoxemia requiring bilevel positive airway pressure and high-flow oxygen. Pleural fluid was frankly purulent, and outside-hospital culture data grew *Gemella sanguinis* and *Escherichia coli*.

**Table 1:** Baseline serum and pleural fluid laboratory results *Reference ranges and diagnostic thresholds are shown for a general clinical context.

Parameter	Specimen	Value	Reference range/diagnostic threshold*
White blood cell count	Serum	18.5 × 10⁹/L	4.0-11.0 × 10⁹/L
C-reactive protein	Serum	250 mg/L	0-10 mg/L
D-dimer	Serum	5,500 ng/mL	<500 ng/mL
pH	Pleural fluid	6.9	Normal pleural fluid approximately 7.60-7.66
Glucose	Pleural fluid	30 mg/dL	Usually >60 mg/dL
Lactate dehydrogenase	Pleural fluid	>1,000 U/L	Elevated; verify against local laboratory reference range

**Figure 1:**
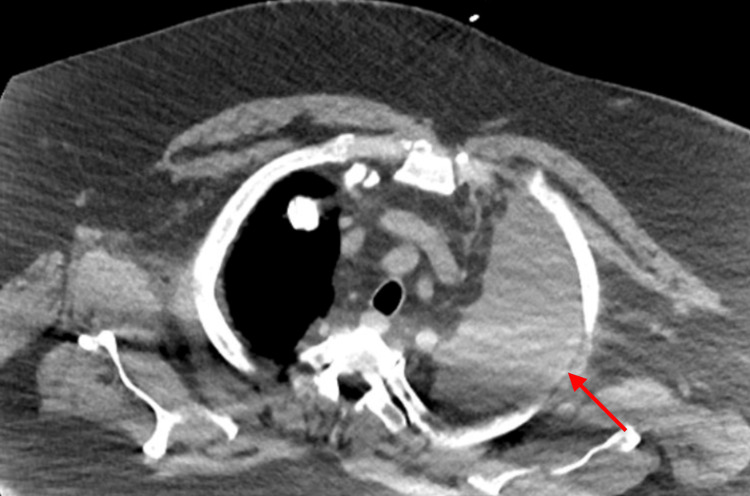
Axial chest CT at presentation demonstrating a large loculated left pleural fluid collection Axial computed tomography (CT) of the chest obtained at presentation demonstrates a large loculated left pleural collection (red arrow), consistent with empyema, with marked compressive atelectatic collapse of the adjacent left lung.

Computed tomography (CT) of the chest demonstrated a large, complex, multiloculated left pleural effusion with compressive atelectasis and mediastinal shift (Figure 1). Because of his severe comorbidity burden, high anesthetic risk, and limited functional reserve, he was considered a poor candidate for general anesthesia and video-assisted thoracoscopic surgery (VATS).

Management was initiated with a 14-French chest tube and intravenous ampicillin-sulbactam. Intrapleural therapy was then started according to the MIST-2 regimen, with tPA 10 mg and DNase 5 mg administered twice daily. After completion of the initial six-dose course, the response remained incomplete: the patient continued to be febrile, the C-reactive protein level had decreased only from 250 to 180 mg/L, and repeat imaging still demonstrated persistent undrained loculations. Because he remained a poor candidate for general anesthesia and VATS, and because no bleeding or other major treatment-related complication had occurred during the initial course, the intrapleural regimen was extended with a second monitored six-dose course of tPA-DNase, for a total of 12 doses administered over six days. Based on culture results and improving clinical stability, he was subsequently transitioned to oral amoxicillin-clavulanate.

**Figure 2:**
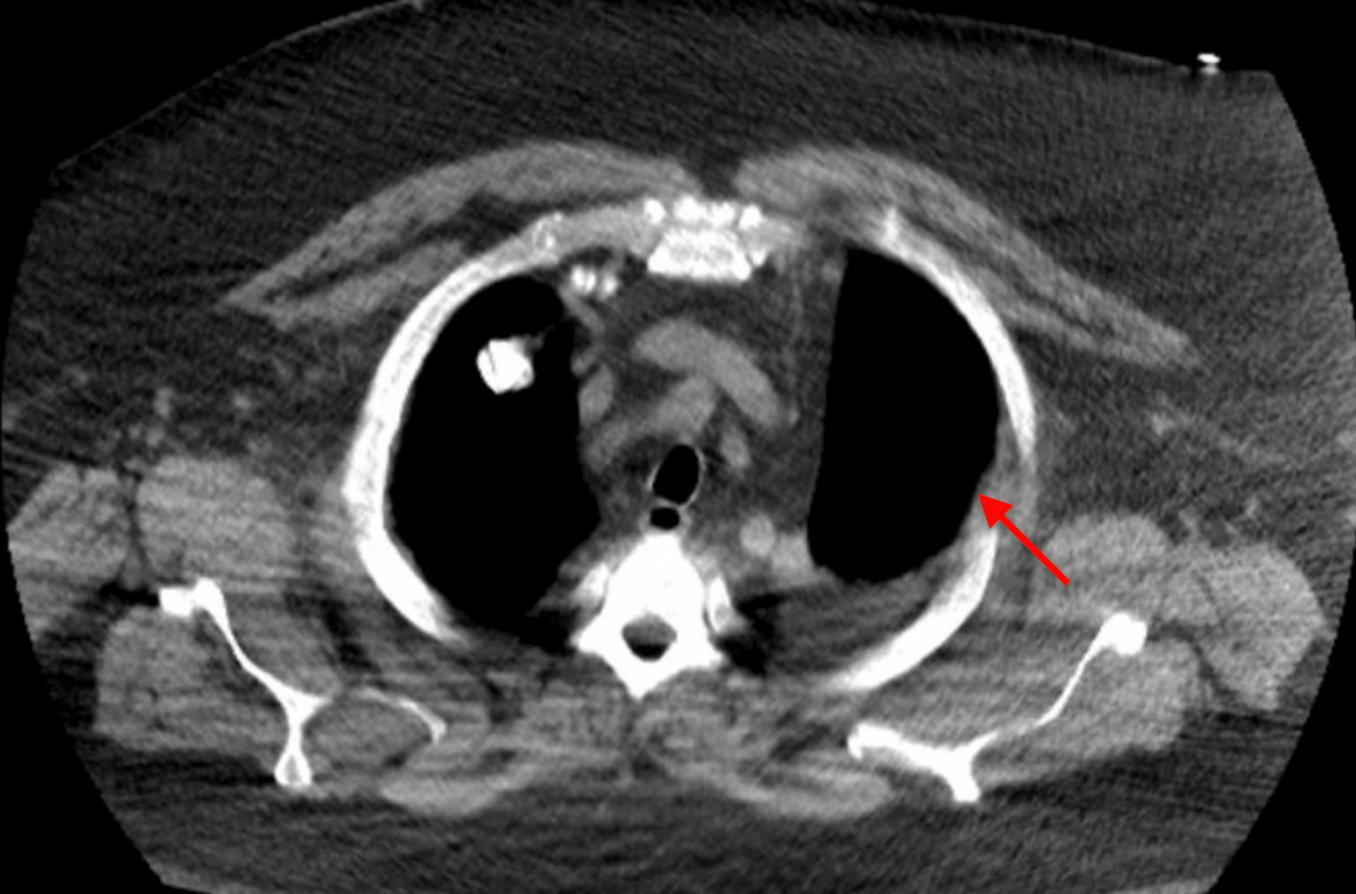
Axial chest CT showing marked interval improvement after extended intrapleural tPA-DNase therapy Follow-up axial computed tomography (CT) of the chest demonstrates marked interval resolution of the previously identified loculated left pleural collection (red arrow), with re-expansion of the left lung after completion of 12 doses of intrapleural tissue plasminogen activator (tPA)-dornase alfa (DNase).

Throughout the extended course, the patient had no intrapleural hemorrhage or other procedure-related complications. With continued treatment, his respiratory status improved substantially, supplemental oxygen was discontinued, and follow-up CT demonstrated near-complete resolution of the pleural effusion with re-expansion of the left lung and no significant residual loculation (Figure 2). After radiographic and clinical improvement, the chest tube was removed, and he was discharged home to complete oral antibiotic therapy.

## Discussion

This case highlights a practical management dilemma not clearly resolved by existing evidence: how to manage a patient with persistent, loculated empyema who has only a partial response to standard intrapleural tPA-DNase therapy and is not a reasonable operative candidate. Although MIST-2 established the clinical role of combined intrapleural tPA-DNase, it did not define how therapy should be adapted when residual loculations persist after completion of the standard six-dose course [4].

Available guidance documents and review literature support the use of tPA-DNase when pleural drainage is inadequate, but they also emphasize ongoing uncertainty regarding optimal dosing, sequencing, and patient selection, particularly beyond the conventional regimen [1,2,6,7]. Observational comparisons suggest that surgery may achieve lower treatment failure rates in selected operative candidates [5,8], whereas randomized comparative data indicate that both intrapleural fibrinolytic therapy and surgery can be feasible strategies in appropriately selected patients [9,10]. However, such data do not resolve management in patients whose comorbidity burden makes VATS high risk or impractical.

In our patient, extension of therapy was guided by three main factors: persistent loculations on repeat imaging after the initial six-dose course, incomplete clinical and inflammatory improvement, and the absence of a safe operative alternative. In this context, a response-guided extension was considered preferable to automatic discontinuation after dose six. This observation should be interpreted cautiously because it derives from a single case; however, it suggests that extension may be feasible in carefully selected patients with persistent radiographic disease, no reasonable surgical option, and no evidence of bleeding during closely monitored treatment.

Bleeding remains the principal safety concern with prolonged intrapleural fibrinolytic therapy. Available evidence suggests that clinically significant bleeding is uncommon but not negligible, underscoring the need for close surveillance throughout treatment [7,8]. In this case, no intrapleural hemorrhage or systemic bleeding event occurred despite the extended course, which supports the feasibility of longer treatment in selected patients under careful monitoring rather than implying established superiority of extended dosing.

The microbiology was also notable. *Gemella *species are uncommon but recognized causes of pleural infection and other invasive disease [9]. In this patient, the coexistence of *G. sanguinis* and *E. coli* further supports the polymicrobial nature that empyema may exhibit and reinforces the importance of aggressive source control together with culture-directed antimicrobial therapy.

## Conclusions

This case highlights extended intrapleural tPA-DNase therapy as a potentially valuable individualized rescue strategy in carefully selected patients with persistent loculated empyema who have an incomplete response to the standard MIST-2 regimen and are poor surgical candidates. While broader evidence is needed before treatment extension can be routinely recommended, this report demonstrates the feasibility of a response-guided extended course, with marked clinical and radiographic improvement and no bleeding complications in a high-risk non-operative patient. In similar settings, extension of therapy may be considered when residual loculations persist, standard therapy is insufficient, and close clinical, radiographic, and bleeding surveillance can be maintained.
